# Mediating role of cognitive flexibility in the relationship between computational thinking and creative problem-solving in esports players

**DOI:** 10.3389/fpsyg.2026.1792475

**Published:** 2026-03-23

**Authors:** Alp Kaan Kilci, Mehmet Yonal, Gokhan Aydin, Ender Ali Uluc, Seyhan Bekir, Ferhat Aktas, Nahit Ozdayi, Orhan Kucuk, Serkan Aksoy

**Affiliations:** 1 Sport Management Department, Faculty of Sport Sciences, Balikesir University, Balikesir, Türkiye; 2 Directorate of Sports Sciences Application and Research Center, Balikesir University, Balikesir, Türkiye; 3 Career Planning Application and Research Center, Gazi University, Ankara, Türkiye; 4 Independent Researcher, Balikesir, Türkiye; 5 Ayvacik Vocational School, Management and Organization Department, Canakkale 18 Mart University, Canakkale, Türkiye; 6 Department of Psychology, Faculty of Science and Letters, Demiroglu Bilim University, Istanbul, Türkiye; 7 Yenice Vocational School, Canakkale 18 Mart University, Canakkale, Türkiye; 8 Faculty of Sport Sciences, Coaching Education Department, Balikesir University, Balikesir, Türkiye; 9 Afyonkarahisar Provincial Directorate of Youth and Sports, Afyon, Türkiye; 10 Ayvacik Vocational School, Canakkale 18 Mart University, Canakkale, Türkiye

**Keywords:** cognitive flexibility, computational thinking, creative problem solving, esport, mediator

## Abstract

**Background:**

The performance of individuals in esports environments is contingent upon the execution of high-level cognitive processes, including the capacity for rapid information processing, strategic decision-making, and the generation of creative solutions in uncertain conditions. The present study examined the mediating role of cognitive flexibility in the effect of esports players’ information processing skills on creative problem-solving.

**Methods:**

This study employed a descriptive and relational design to investigate the relationships between computational thinking (CT), creative problem-solving (CPS), and the mediating role of cognitive flexibility (CF) among esports players. Research data were collected via Google Forms from 438 esports players selected through purposive sampling at the Digital Youth Center in Balıkesir. The research measures comprised the Computational Thinking Scale, the Cognitive Flexibility Scale, and the Creative Problem-Solving Scale. Data analysis was performed using SPSS 27.0, where normality was confirmed through Skewness and Kurtosis values within the ±1.5 range. The mediation model was tested using Hayes’ PROCESS macro (Model 4) with a bootstrapping method (5,000 resamples at a 95% confidence interval), evaluating the indirect effects of CT on CPS through CF.

**Results:**

The present study demonstrated a positive and significant relationship between information computational thinking (CT) and both cognitive flexibility (CF) (*r* = 0.628, *p* < 0.01) and creative problem solving (CPS) (*r* = 0.614, *p* < 0.01). Also found that there was a positive correlation between CF and CPS (*r* = 0.669, *p* < 0.01). In the mediation model, computational thinking significantly predicted CF (*a*: *b* = 0.4425, *p* < 0.001); CF also significantly predicted CPS (*b*: *b* = 1.2066, *p* < 0.001). The total effect (*c*: *b* = 1.1165, *p* < 0.001) decreased when the mediating variable (CF) was included, yet it remained significant (*c*′: *b* = 0.5826, *p* < 0.001). Therefore, it can be concluded that mediation has occurred. The indirect effect was found to be significant, with a value of *b* = 0.5339. This is due to the 95% bootstrap confidence interval [0.3889, 0.6836] not including zero.

**Conclusion:**

Computational thinking enhances creative problem-solving in esports players, both directly and indirectly through cognitive flexibility. The findings of this study indicate that the integration of computational thinking skills with cognitive flexibility into training processes could enhance creative and situationally adaptable decision-making capacity, thereby supporting esports performance.

## 1 Introduction

Nowadays, sporting performance requires not only a high level of physical capacity, but also the ability to adapt to rapidly changing game dynamics, make the right decisions under pressure, and develop creative solutions in unexpected situations (Lin et al., 2019; Sagoo, 2017; Welsh et al., 2018). Success in sports disciplines where environmental uncertainty is high and explicit skills are paramount cannot be explained solely by motor skills; it also shows a strong correlation with advanced cognitive processes (Memmert, 2015; Raab, 2012). In this context, the ability to generate unique, functional and context-appropriate solutions to problems encountered by players on the field stands out as one of the key components of sporting success. Furthermore, players’ psychological state and performance levels are closely related to their problem-solving skills (Lin et al., 2025). This requirement becomes even more pronounced in esports, where performance is predominantly determined by rapid information processing, strategic decision-making, and adaptive problem-solving under extreme time pressure. This is due to the fact that esports is an inherently dynamic field, requiring cognitive skills that are capable of adapting swiftly to change. The findings of neurocognitive research have indicated that individuals who engage in regular participation in competitive esports games exhibit superior cognitive abilities in comparison to those who do not actively engage in such activities. (Bediou et al., 2018; Kowal et al., 2018). Chen et al. (2014) proved that there is a strong link between creative thinking and cognitive flexibility. They showed that people with creative thinking skills can respond more flexibly and adaptively to complex real-life situations. Similarly, Li et al. (2023) showed that cognitive flexibility is a key cognitive ability that promotes success, particularly due to its ability to generate different solutions in new and challenging situations.

Creative problem-solving skills are a fundamental ability that manifests itself in every area of an individual’s life; it is present in all activities, ranging from simple everyday situations to complex processes (Amran et al., 2019). In the context of sporting competition, the term “decision-making” signifies an individual’s capability to reach the optimal decision when facing challenges or demands imposed by an opponent, to diverge from established tactics if required, and to devise novel strategies within the flow of the sporting event (Memmert and Roth, 2007). Accordingly, Furley and Memmert (2018) reported a positive association between creative problem-solving skills and players’ tactical intelligence, game-reading abilities, and overall performance levels. Consequently, the study of cognitive mechanisms such as creative problem solving in players constitutes a significant research domain within the field of sports science. It has been demonstrated that regular exercise has a positive effect on creative problem-solving skills (Díaz-Rodríguez and Pérez-Córdoba, 2024; Shariati et al., 2025; King et al., 2024) These findings necessitate an explanation of the cognitive mechanisms through which creative problem-solving develops in a sporting environment; at this point, computational thinking, which structures and systematizes problem-solving processes, appears to offer an explanatory framework (Wing, 2006; Grover and Pea, 2013).

In recent years, a particular concept has assumed a prominent role in the field of cognitive process analysis: information processing thinking skills. Computational thinking is defined as a cognitive process involving the skills of breaking down a problem into parts, abstracting it, and developing algorithms to solve the problems encountered (Wing, 2006; Brennan and Resnick, 2012). It is acknowledged that computational thinking is not confined to technical domains; it contributes to creative problem-solving processes in daily life and across different disciplines (Grover and Pea, 2013). Computational thinking, regarded as a constituent element of problem solving, has become an increasingly significant skill in recent years (Wing, 2006). It is evident that the computational thinking is a significant cognitive framework that can facilitate creative problem-solving abilities (Costa et al., 2017; Sung et al., 2017; Yadav et al., 2011; Maharani et al., 2019). When considered in the context of esports, computational thinking can be conceptualized as the player’s ability to analyze complex game situations, abstract relevant information, and formulate algorithmic decision strategies in real time. These processes have the capacity to exert a substantial influence on in-game decision-making skills, particularly within the context of esports, where elevated cognitive demands are imperative. In this regard, Dye et al. (2009) and Kowal et al. (2018) reported that individuals who regularly play video games demonstrate significantly higher levels of computational thinking compared to non-players, suggesting that these cognitive advantages may contribute to esports performance.

The relationship between computational thinking and creative problem solving can be supported by the concept of cognitive flexibility. It is thought that the fundamental reason for this stems from the definition of cognitive flexibility as the capacity of individuals to adapt to changing circumstances, develop different perspectives and alter their thinking strategies when necessary (Dennis and Vander Wal, 2010; Diamond, 2013). A high level of cognitive flexibility is also required in esports due to time pressure, opponent behavior and constantly changing tactical situations. Additionally, esports players must constantly change their strategy, react to unpredictable opponent behavior, and adapt to rapidly evolving in-game situations. This renders cognitive flexibility a pivotal component of success in esports. Green et al. (2012) and Toth et al. (2019) reported that individuals who engage in esports demonstrate superior task-switching abilities compared to non-players. This finding serves to underscore the high levels of cognitive flexibility exhibited by individuals who engage in esports gaming. Vestberg et al. (2012) suggested that players with high cognitive flexibility can adapt more quickly to new conditions, manage the learning process from mistakes more effectively, and generate creative solutions on the spot.

The capacity to computational thinking is known to facilitate the analysis of problems from diverse perspectives, leading to the development of alternative solutions (Barr and Stephenson, 2011; Shute et al., 2017). The development of this skill is hypothesized to be influenced by cognitive flexibility. It is evident that cognitive flexibility plays a pivotal role in the realm of creative problem solving within these cognitive processes (Deak, 2003; Ionescu, 2012; Diamond, 2013; Zabelina and Robinson, 2010). In other words, the effect of computational thinking on creative problem solving may be developed through cognitive flexibility.

However, despite the growing body of research examining computational thinking, cognitive flexibility, and creative problem-solving independently, there is a notable lack of integrative models that explain how these cognitive constructs interact within athletic populations. Most existing studies have either focused on educational contexts (Jenny et al., 2024) or investigated direct associations, thereby overlooking the underlying cognitive mechanisms that may mediate these relationships. Nevertheless, it should be noted that, like traditional sports, eSports require on-the-field performance. As in traditional sports, this in-sport performance places great importance on cognitive flexibility (Imanian et al., 2024; Valls-Serrano et al., 2022). These studies have shown that decision-making, fluid intelligence, attention control, inhibition, working memory, and cognitive flexibility predict success in games. According to the results of this study, individuals with greater cognitive flexibility tend to be more successful in games, and this success correlates with their performance in the game (Valls-Serrano et al., 2022). Despite the scarcity of these studies, it can be concluded that increasing intermediary studies on cognitive flexibility is important. Empirical evidence examining the mediating role of cognitive flexibility in the relationship between computational thinking and creative problem-solving among players especially within performance-oriented and dynamically complex environments such as esports remains scarce.

The theoretical framework of the study is based on the premise that cognitive flexibility, particularly in an active, dynamic field such as esports where one constantly encounters different situations and must make snap decisions, is a fundamental mechanism in the process of generating creative and situation-appropriate solutions to problems encountered through information processing thinking skills. The ability to break down problems into smaller components and then generate solutions through the application of computational thinking skills is a key factor in the development of cognitive flexibility. This, in principle, enables players to reassemble these components and select the most appropriate option in situations that require a rapid response. In this context, cognitive flexibility is theoretically considered to be a mediating variable that demonstrates the effect of computational thinking skills on creative problem solving. In this context, two research questions (RQs) have been formulated:

*RQ1*: Does computational thinking ability predict creative problem-solving ability?

*RQ2*: Does cognitive flexibility mediate the role of relations in the computational thinking ability on creative problem-solving ability?

## 2 Materials and methods

### 2.1 Research design and participants

Purposeful sampling was preferred in the participant selection process for this research. Regarding statistical representativeness, Sekaran (2003) asserts that a sample size of 384 is adequate for a 5% confidence interval in populations of considerable size. The sample size of 438 individuals reached in this research meets the relevant scientific threshold and supports the generalizability of the analyses. Consequently, the sample size of the research (438) indicates that the results can be generalized.

This research is based on a comprehensive methodological framework combining descriptive and correlational survey models to determine the status of the variables and explain the complex interactions between them. The primary focus of the research is an examination of the relationship between Computational Thinking (CT) and Creative Problem Solving (CPS) in the context of esports. Furthermore, a mediation model was applied to analyze the mediating role of Cognitive Flexibility (CF) in this interaction. The hypotheses, which form the core of the research framework and represent the fundamental assumptions of the research, have been formulated as follows:

*H1*: There is a statistically significant relationship between esports players’ computational thinking skills, cognitive flexibility, and creative problem-solving abilities.

**Figure 1:**
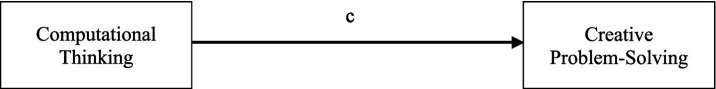
Total effects of computational thinking on creative problem-solving.

*H2*: Esports players’ computational thinking skills significantly predict their creative problem-solving abilities (Figure 1).

**Figure 2:**
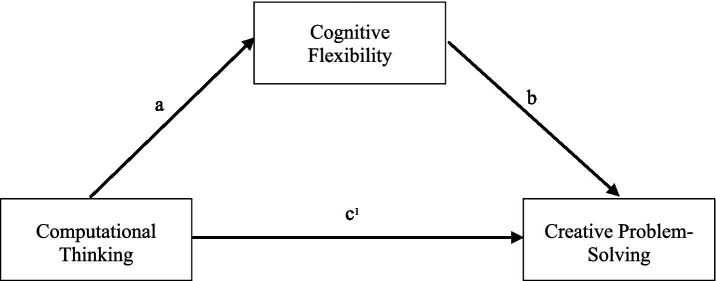
The mediation model proposes that computational thinking indirectly predicts creative problem-solving through cognitive flexibility.

*H3*: Cognitive flexibility in sport mediates the relationship between computational thinking and creative problem-solving (Figure 2).

### 2.2 Data collection tools

The data collected in this research was gathered via Google Forms from players participating in esports games at the Digital Youth Centre in Balıkesir, where esports games are played, as a purposeful sampling method was employed both online and in person. As data collection tools, researchers utilized a personal information form, computational thinking scale, cognitive flexibility scale, and creative problem-solving scale.

#### 2.2.1 Personal information form

The personal information form created by researchers contains questions about the esports players’ gender, age, the year they actively played esports games (as a year), and the esports game they played.

#### 2.2.2 Computational thinking scale

The Computational Thinking Scale, developed by Tsai et al. (2021), aims to measure thinking processes related to computational thinking. The scale consists of 19 items rated on a 5-point Likert scale ranging from “Strongly agree (5)” to “Strongly disagree (1).” The Turkish adaptation of the computational thinking scale was adapted by Gök and Karamete (2023), and its five-dimensional structure has been validated. The scores obtained on the scale range from 19 to 95. Scale consists of five sub-dimensions: scaling, abstraction, decomposition, algorithmic thinking, evaluation and generalization. The internal consistency coefficients of the factors comprising the scale were determined as *α* = 0.83 for abstraction, *α* = 0.81 for differentiation, *α* = 0.74 for algorithmic thinking, *α* = 0.75 for evaluation, and *α* = 0.77 for generalization.

#### 2.2.3 Cognitive flexibility scale

The Cognitive Flexibility Scale (CFS), introduced to the literature by Martin and Rubin (1995) and adapted into Turkish by Çelikkaleli (2014), was used in the study to determine participants’ levels of cognitive flexibility. This scale, which has a one-dimensional structure, consists of 12 items on a 6-point Likert scale (1: Strongly Disagree, 6: Strongly Agree). Items 2, 3, 6 and 10 on the scale are reverse-scored, and the total score ranges from 12 to 72. The high scores obtained indicate the individual’s high cognitive flexibility capacity. In the validity and reliability analyses conducted by Çelikkaleli (2014), the scale’s internal consistency coefficient was reported as 0.74, the test–retest correlation as 0.98, and the split-half reliability as 0.77. The scale demonstrated an internal consistency coefficient of 0.80 in its original form.

#### 2.2.4 Creative problem-solving scale

The creative problem-solving scale developed by Lin (2010) and adapted into Turkish by Bulut et al. (2018) was used to assess participants’ creative problem-solving skills. The 40-item scale, structured as a five-point Likert scale, consists of five sub-dimensions: convergent thinking, divergent thinking, environment, motivation, and general knowledge. In the adaptation research, the internal consistency coefficient α was determined to be 0.85 for the scale as a whole; the reliability coefficients for the subscales were found to be 0.78 for convergent thinking, 0.79 for divergent thinking, 0.88 for environment, 0.73 for motivation, and 0.77 for general knowledge.

### 2.3 Ethical approval

This research has been ethically approved by the Ethics Committee of the Faculty of Social and Human Sciences at Balıkesir University under decision number 2025/05–28. Furthermore, informed consent forms were obtained from participants prior to answering the questions.

### 2.4 Statistical analysis

The analysis of the research data was conducted using SPSS 27.0 statistical software. The exported data from the online environment was initially saved as an Excel file. Thereafter, the data set was transferred to SPSS. Basic statistical operations were performed on the cleaned data set in order to test the research hypotheses. In addition to the descriptive statistics employed in the study (e.g., percentage, frequency, mean, and standard deviation), Skewness and Kurtosis values were examined to ascertain whether the data followed a normal distribution. The analyses revealed that the Skewness and Kurtosis values ranged between −1.5 and +1.5. The findings indicate that the data exhibit a normal distribution and are suitable for the use of parametric tests, in line with the criteria proposed by Tabachnick et al. (2007).

To test the mediating role of cognitive flexibility in the relationship between computational thinking and creative problem solving, the PROCESS macro (Model 4) developed by Hayes (2022) was used. The statistical significance of indirect effects was analyzed using the bootstrapping method with 5,000 resamples and a 95% confidence interval (CI). According to Hayes’ methodology, the fact that the confidence interval values (LLCI and ULCI) for the indirect effect of the independent variable on the dependent variable did not include zero was taken as the basic criterion for accepting that the mediating effect was statistically significant.

## 3 Results

The results from the study first present descriptive statistics in Table 1. Skewness and kurtosis values were also analyzed to determine normal distribution. No multicollinearity issues were detected, and the data were found to be normally distributed. The summary of the findings, including the results of the Pearson correlation analysis between the variables, is also presented in Tables 1, 2.

**Table 1:** Demographic characteristics of the esports players.

Demographic characteristics	Categories	Frequency (*n*)	Percentage (%)
Gender	Female	138	31.5
	Male	300	68.5
Age	18	75	17.1
	19	58	13.2
	20	90	20.5
	21	72	16.4
	22+	143	32.6
Esport branch	FPS	233	53.2
	MOBA	205	46.8
Esport experience	1–3 years	102	23.3
	4–6 years	229	52.3
	7 + years	107	24.4

FPS, First Person Shooter; MOBA, Multiplayer Online Battle Arena.

**Table 2:** Descriptive statistics, linearity, normality, multicollinearity, and correlations.

Variables	Mean	SD	Skew.	Kurt.	VIF	CI	1	2	3
1-Computational thinking	62.95	16.56	−0.48	0.12		1	1		
2-Cognitive flexibility	43.85	11.80	−0.33	0.23	1.58	9.00	0.62^**^	1	
3-Creative problem-solving ability	130.42	30.37	0.53	0.42	1.58	10.70	0.61^**^	0.66^**^	1

^**^p < 0.01.

A demographic analysis of the group of esports players participating in the study showed that 31.5% (*n* = 138) of the players were female and 68.5% (*n* = 300) were male. With regard to age distribution, the players were as follows: The age of the participants ranged from 17 to 22 years old, with 18-year-olds constituting the largest age group at 17.1% of the total sample (*n* = 75), followed by 19-year-olds (13.2%, *n* = 58), 20-year-olds (20.5%, *n* = 90), 21-year-olds (16.4%, *n* = 72), and those aged 22 and above (32.6%, *n* = 143). Regarding the esports genre, 53.2% of players (*n* = 233) stated that they play FPS games, while 46.8% (*n* = 205) stated that they play MOBA games. The duration of players’ esports experience was found to be 1–3 years (23.3%, *n* = 102), 4–6 years (52.3%, *n* = 229) and 7 years and above (24.4%, *n* = 107) (Table 1).

Jondeau and Rockinger (2003) observed that, Variance Inflation Factor (VIF) and Condition Index (CI) values to assess the presence of multicollinearity. The findings of this article revealed that the values were acceptable (Montgomery et al., 2021). Consequently, no multicollinearity was identified between the variables in this study. Therefore, no problems were encountered when reporting with the HAYES PROCESS (Hayes, 2022).

The analyses revealed a positive and significant relationship between Computational Thinking and Cognitive Flexibility (*r* = 0.62, *p* < 0.01) and Creative Problem Solving (*r* = 0.61, *p* < 0.01). A positive and significant relationship was also observed between Cognitive Flexibility and Creative Problem Solving (*r* = 0.66, *p* < 0.01) (Table 2).

### 3.1 Mediating role of cognitive flexibility

**Figure 3:**
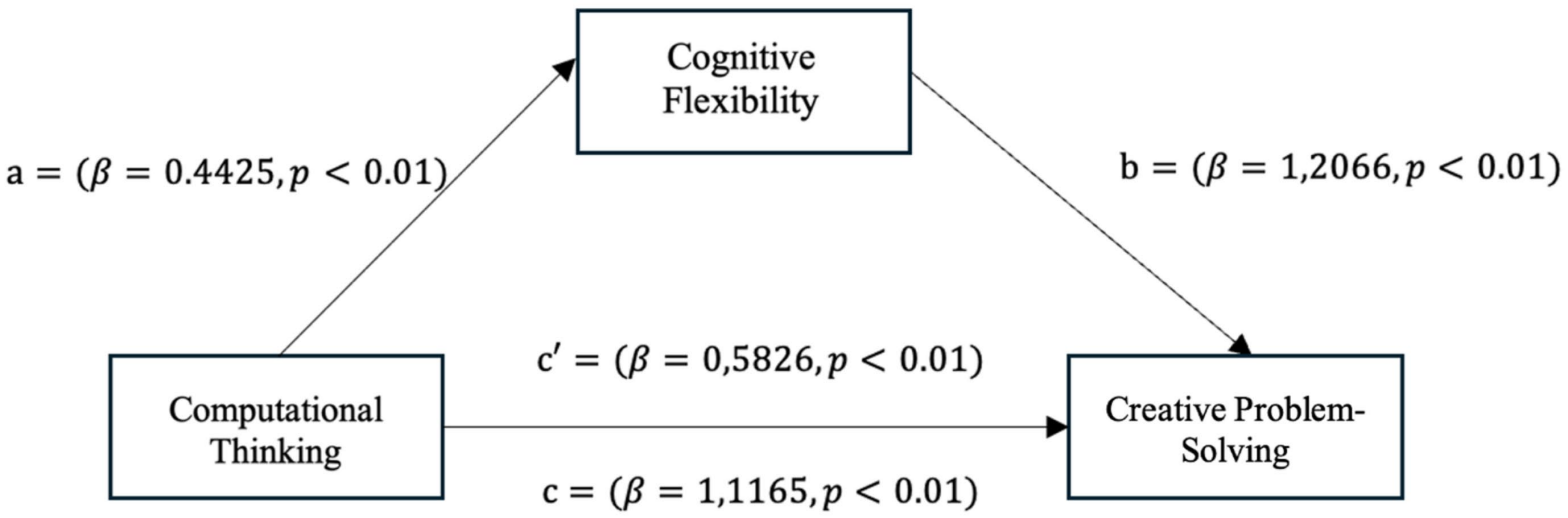
Model of the mediating role of cognitive flexibility in the relationship between computational thinking and creative problem solving.

The results regarding the mediating role of cognitive flexibility is shown in Table 3 and Figure 3.

**Table 3:** Mediating model coefficients.

Predictors	2-cognitive flexibility			3-Creative Problem-Solving Ability		
	Path	*b*	*p*	Path	*b*	*p*
1-Computational thinking	*a*	0.44	0.00	*c′*	0.58	0.00
2-Cognitive flexibility		–	–	*b*	1.21	0.00
Constant	*i*_1_	15.89	0.00	*i*_2_	40.25	0.00
	*R* = 0.617, *R*^2^ = 0.381			*R* = 0.709, *R*^2^ = 0.503		
	*F*(1, 436) = 156.78, *p* < 0.001			*F*(2, 435) = 88.88, *p* < 0.001		

To test direct and indirect paths in the study, PROCESS Macro v5.0 and Model 4 were used with 5,000 bootstrap samples and a 95% confidence interval (Hayes, 2022). At this point, our initial analysis examined that we examined the direct pathways of computational thinking on cognitive flexibility and creative problem-solving ability (*a*₁, pathways, Table 3) and the pathway from cognitive flexibility to creative problem-solving ability (path *b*₁). Supporting the proposed direct effects, computational thinking (CT) was found to be significantly associated with cognitive flexibility (CF). All the paths are indicated in Figure 3.

To analyze the relationship between computational thinking and creative problem-solving ability, a 5,000-resample bootstrap method was used to test the mediation hypothesis. The analysis indicated that the relationship between computational thinking and creative problem-solving ability is indirect and mediated by cognitive flexibility. This indirect effect is statistically significant because the 95% bootstrap confidence interval does not include zero (indirect effect = 0.5339; 95% CI [0.3889, 0.6836]), indicating that this relationship occurs indirectly through cognitive flexibility. Additionally, all paths were positive and in the expected paths. The effects of computational thinking on cognitive flexibility and of cognitive flexibility on creative problem-solving ability were both positive and significant (*b* = 0.44, *p* < 0.001 and *b* = 1.21, *p* < 0.001, respectively). These results suggest that cognitive flexibility significantly mediates the relationship between computational thinking and creative problem-solving ability (Table 4).

**Table 4:** Bootstrapping process of partial model.

**Pathways**	**Effect**	**SE**	***p***	**LLCI**	**ULCI**
Direct effects Negative problem orientation → Social problem-solving	–3.26	0.32	0.00	–3.894	–2.632
Total effects Negative problem orientation → Social problem-solving	–2.28	0.43	0.00	–3.134	–1.443
Indirect effect	**Effect**	**BootSE**		**BootLLCI**	**BootULCI**
Negative problem orientation → Cognitive flexibility → Social problem-solving	0.97	0.25		0.4805	1.485

## 4 Discussion

This research examines the relationship between computational thinking, cognitive flexibility and creative problem-solving skills, and the mediating role of cognitive flexibility. The findings provide substantial support for the proposed hypotheses and contribute to a more nuanced understanding of the role of cognitive flexibility, one of the underlying cognitive mechanisms of creative problem solving, in this skill. Furthermore, it demonstrates that creative problem solving cannot be explained by a single cognitive skill; rather, it arises from the interaction of analytical and adaptive cognitive processes.

The findings of this study indicated the presence of a significant and positive correlation between computational thinking, cognitive flexibility, and creative problem-solving ability. Consequently, H1 was accepted. Computational thinking, cognitive flexibility and creative problem-solving skills are complementary cognitive competencies, and the variable structure of information processing and cognitive flexibility enhances creative problem-solving skills. This is especially the case when confronted with complex and ambiguous issues, where individuals require both structured thinking skills and cognitive flexibility. A review of the extant literature reveals findings that are consistent with the research undertaken. Diamond (2021) states that cognitive flexibility provides individuals with the capacity for adaptation, enabling them to utilize different cognitive and psychological strategies, particularly in problem-solving processes involving uncertainty. This, in turn, provides behavioral flexibility and facilitates the generation of solutions to encountered problems. Bedel and Ulubey (2015) found that cognitive flexibility and information processing skills are interrelated, while Hassinger-Das et al. (2018) found that flexibility is a necessary concept for problem solving, while Lee et al. (2024) demonstrated that cognitive flexibility enables the development of different strategies and the ability to switch between tasks, thereby positively influencing problem-solving skills. Researches have stated that cognitive flexibility, as a fundamental component of executive functions, is critical in enabling individuals to adapt to changing conditions, make flexible transitions between concepts, and revise their existing strategies to achieve defined behavioral goals (Dajani and Uddin, 2015; Hohl and Dolcos, 2024). It is posited that this approach affords individuals a strategic margin for maneuver, particularly in circumstances where conventional solutions prove to be ineffective. Consequently, when individuals encounter a complex problem, their ability to move away from rote learning and transition to a more dynamic and analysis-based logical reasoning process demonstrates the decisive impact of cognitive flexibility on problem-solving success. These results support findings that emphasize how higher-level cognitive skills work together in creative and problem-solving processes.

It was found that computational thinking skills were a significant predictor of creative problem-solving ability, and H2 was accepted. This situation shows that esports players can use computational thinking skills such as abstraction, decomposition and algorithmic reasoning to structure complex variables in dynamic gaming environments. This algorithmic approach provides a technical foundation that enables esports players to generate creative strategies by converting errors into real time data, even under high cognitive load, thus transcending traditional gaming practices. Therefore, computational thinking skills play a critical role in accelerating strategic decisions through the analysis and abstraction of in-game dynamics. A review of the literature reveals that other studies support the findings of this study, showing that computational thinking has a positive effect on creative problem-solving skills (Kobsiripat, 2015; Murcia et al., 2020; Pérez Poch et al., 2016). Furthermore, computational thinking is based on breaking down complex problems into smaller parts, designing systematic solution methods for these sub-parts, and demonstrating an algorithmic approach throughout all these stages (Wing, 2006; Denning, 2017; Weintrop et al., 2016). The difficulty the mind feels when faced with a complex and large problem is alleviated by breaking the process down into smaller, more manageable parts (decomposition). Additionally, it is known that cognitive flexibility plays a critical role in cognition, creative problem solving, and thinking outside the box, encompassing problem solving and the trial-and-error process (Stevens, 2009; Peters and Crone, 2014).

The essence of esports is all about acting rapidly and making judgements in moments. This involves swiftly assessing intricate in-game scenarios to break them down into bite-sized, manageable components. This systematic way of thinking enables the esports player to devise instant, original, and innovative algorithms (game plans) based on the opponent’s moves, rather than simply repeating existing game tactics. This is because as the problem is broken down into smaller parts, it becomes much easier to develop different and innovative perspectives for each part.

According to the research results, cognitive flexibility was found to play a significant mediating role in the relationship between information processing thinking and creative problem solving, and H3 was accepted. The continued direct impact of computational thinking on creative problem-solving shows that this skill makes an independent contribution. Meanwhile, the indirect impact occurs through cognitive flexibility, suggesting that flexible cognitive processes are a vital part of this relationship. In this context, it can be said that computational thinking supports creative problem-solving by facilitating flexible cognitive processes, such as developing different perspectives, changing strategies and evaluating alternative solutions, as well as by employing linear and analytical means. This situation shows that esports players use the cognitive flexibility they develop within the game to surprise their opponents with creative moves. The continued direct impact of computational thinking on creativity demonstrates that systematic thinking discipline is a power for esports players; while the indirect impact occurs through cognitive flexibility, revealing that the capacity to adapt to dynamic changes within the game elevates this process to a much higher level. These findings support Diamond (2021) view that cognitive flexibility allows for strategic adaptability in uncertain situations. The systematic approaches of esports players within the game are not merely an analytical process of computational thinking (Wing, 2006); they are also a dynamic process that interacts with cognitive flexibility, as emphasized by Bedel and Ulubey (2015). The presence of indirect effects demonstrates that esports players can produce creative moves that surprise their opponents by combining algorithmic thinking discipline with flexible mental processes. This enables them to utilize the cross-task flexibility advantage mentioned by Lee et al. (2024) to generate creative solutions to problems.

## 5 Conclusion

This research has revealed that the computational thinking, cognitive flexibility and creative problem-solving skills of esports players interact with one another. The findings show that the ability to generate creative solutions in esports stems from combining computational thinking with cognitive flexibility. Computational thinking enables players to break down complex problems into meaningful parts and build systematic strategies based on these parts, even in the chaotic and fast-paced flow of the game. However, the study’s notable outcome is that cognitive flexibility facilitates the transformation of analytical thoughts into creativity. This demonstrates that esports players do not simply adhere to pre-determined plans but rather update their strategies swiftly in response to the evolving conditions of the game, executing moves that their opponents cannot anticipate. In summary, the ability to think analytically, provided by information processing, and the ability to generate different solutions, brought about by cognitive flexibility, are important criteria that enable esports players to produce original solutions, even under intense pressure.

## 6 Limitations

This research has several limitations. One is that the sample consists of esports players. Another is that it was collected only from a specific region within a particular city. Also, data were collected using self-report questionnaires. This constitutes a significant limitation of the study. Finally, the study is cross-sectional and does not establish causality. Instead of causality, mediation and prediction are emphasized. AVE and SFL values were not calculated for the scales, which represents a limitation of the study.

## Data Availability

The raw data supporting the conclusions of this article will be made available by the authors, without undue reservation.
